# Exome genotyping and linkage analysis identifies two novel linked regions and replicates two others for myopia in Ashkenazi Jewish families

**DOI:** 10.1186/s12881-019-0752-8

**Published:** 2019-01-31

**Authors:** Claire L. Simpson, Anthony M. Musolf, Qing Li, Laura Portas, Federico Murgia, Roberto Y. Cordero, Jennifer B. Cordero, Bilal A. Moiz, Emily R. Holzinger, Candace D. Middlebrooks, Deyana D. Lewis, Joan E. Bailey-Wilson, Dwight Stambolian

**Affiliations:** 1 Department of Genetics, Genomics and Informatics and Department of Ophthalmology, University of Tennessee Health Science Center, 71 S. Manassas Room 417, Memphis, TN 38163 USA; 2 Computational and Statistical Genomics Branch, National Human Genome Research Institute, National Institutes of Health, 333 Cassell Dr., Suite 1200, Baltimore, MD 21224 USA; 3 Department of Ophthalmology, University of Pennsylvania, Rm. 313, Stellar Chance Labs, 422 Curie Blvd, Philadelphia, PA 19104 USA

**Keywords:** Myopia, Genetic linkage, Family studies

## Abstract

**Background:**

Myopia is one of most common eye diseases in the world and affects 1 in 4 Americans. It is a complex disease caused by both environmental and genetics effects; the genetics effects are still not well understood. In this study, we performed genetic linkage analyses on Ashkenazi Jewish families with a strong familial history of myopia to elucidate any potential causal genes.

**Methods:**

Sixty-four extended Ashkenazi Jewish families were previously collected from New Jersey. Genotypes from the Illumina ExomePlus array were merged with prior microsatellite linkage data from these families. Additional custom markers were added for candidate regions reported in literature for myopia or refractive error. Myopia was defined as mean spherical equivalent (MSE) of -1D or worse and parametric two-point linkage analyses (using TwoPointLods) and multi-point linkage analyses (using SimWalk2) were performed as well as collapsed haplotype pattern (CHP) analysis in SEQLinkage and association analyses performed with FBAT and rv-TDT.

**Results:**

Strongest evidence of linkage was on 1p36(two-point LOD = 4.47) a region previously linked to refractive error (*MYP14*) but not myopia. Another genome-wide significant locus was found on 8q24.22 with a maximum two-point LOD score of 3.75. CHP analysis also detected the signal on 1p36, localized to the *LINC00339* gene with a maximum HLOD of 3.47, as well as genome-wide significant signals on 7q36.1 and 11p15, which overlaps with the *MYP7* locus.

**Conclusions:**

We identified 2 novel linkage peaks for myopia on chromosomes 7 and 8 in these Ashkenazi Jewish families and replicated 2 more loci on chromosomes 1 and 11, one previously reported in refractive error but not myopia in these families and the other locus previously reported in the literature. Strong candidate genes have been identified within these linkage peaks in our families. Targeted sequencing in these regions will be necessary to definitively identify causal variants under these linkage peaks.

**Electronic supplementary material:**

The online version of this article (10.1186/s12881-019-0752-8) contains supplementary material, which is available to authorized users.

## Background

Myopia is a common, complex trait with both genetic and environmental factors influencing risk [1, 2]. As rates of myopia have been increasing rapidly in many parts of the world, myopia is one of the most preventable forms of blindness that imposes significant socio-economic costs. Recent genomewide association studies (GWAS) have identified a number of loci associated with the risk of developing refractive errors [3–9] but so far few causal variants have been identified. Whole exome sequencing (WES) has been used in a number of traits to identify causal variants that modify the risk of developing traits and diseases and although this can be an attractive approach in phenotypes that are relatively uncommon, the challenges for identifying which variants are truly causal in a common trait like myopia are much greater. Population-based designs can be difficult to analyze and interpret for WES data and the sample size requirements can be prohibitive, especially as the cost of sequencing remains relatively high. Family-based study designs have several advantages over population-based studies, especially when focusing on rare variants, as these may be enriched within a family even if they are rare in the population and require lower numbers of individuals to retain sufficient power. This approach has been used successfully to identify genes increasing risk for pathogenic or “high” myopia (mean spherical equivalent (MSE) < − 6 diopters (D)) [10–12]. Family-based linkage studies using sparse panels of genetic markers (microsatellites and common single nucleotide polymorphisms (SNPs)) have identified regions of the genome likely to be harboring high-risk rare variants contributing to non-pathogenic myopia (MSE < −1D) in highly aggregated families [13–22] but the causal variants responsible for these results have not yet been identified. Exome-focused arrays such as the Illumina ExomePlus array provide an inexpensive way of surveying variation in the coding regions of the genome, with content more targeted at coding variation. This study uses dense exome array genotype data to attempt to narrow in on genes with rare variants that strongly increase risk of myopia in our highly-aggregated Ashkenazi Jewish families from the Penn Family Study.

## Methods

### Study design

#### Patient recruitment and genotyping

Genotype data were available for 527 Ashkenazi Jewish individuals (64 extended families) selected due to their strong information content for linkage studies of myopia from among the 105 Ashkenazi Jewish families included in the Penn Family Study. Details of the recruitment of these families has been previously described [14]. This study followed the tenets of the Declaration of Helsinki and informed consent was obtained from all subjects after explanation of the nature of the study and any potential consequences. This study was approved by the institutional review boards of the University of Pennsylvania and the National Human Genome Research Institute. All subjects were genotyped with the Illumina ExomePlus array by the Center for Inherited Disease Research (CIDR) at Johns Hopkins University.

### Quality control

CIDR standard quality control procedures were applied to the entire dataset. Blind duplicates and HapMap controls were distributed across plates for concordance checking. Cases and controls were evenly distributed across plates, but family members were kept on the same plate. Samples with suspected mixtures or unusual X and Y patterns or gender mismatch identified and dropped before release. SNP clustering was performed on all SNPs in project and SNP genotypes with genotype quality (GC) score less than 0.15 recoded as missing genotypes. Autosomal SNPs with less than 85% call rate, cluster separation of less than 0.3 and heterozygote rate greater than 80% were dropped. Subsets of SNPs manually reviewed are detailed in Supplementary Methods and details of SNPs not released due to technical failure can be found in Additional file 1: Table S1.

After receiving data from CIDR, additional quality control measures were applied. Genotype and phenotype data were combined and an additional 85 ungenotyped individuals were added to the pedigrees to complete family relationships. Detailed Mendelian error checking was performed in Sib-pair [23], sex discrepancies were calculated in PLINK [24] and samples which did not appear sufficiently matched to their recorded sex were dropped. Any unexpected duplicate samples were identified using PREST-PLUS [25] and one of the duplicate pair dropped. SNPs with > 1 errors in blind duplicates or HapMap controls were dropped and SNPs with > 1 Mendelian error after correction of pedigree relationships were also removed. Batch effects were tested for using a homogeneity test of minor allele frequency for each SNP on each plate compared to all other plates. [26] We averaged these statistics over all SNPs to determine how the plates deviated from each other in [27] PLINK. Heterozygosity rates across samples were checked and outlier samples excluded. Examination of samples for chromosomal abnormalities was performed and problematic samples identified. Autosomal SNPs with sex difference in allelic frequency > 0.2, sex difference in heterozygosity > 0.3 were also excluded. Variants monomorphic in the study were also excluded.

We did not filter SNPs based on Hardy-Weinberg equilibrium (HWE), instead SNPs that were not in HWE were flagged. All significant and suggestive SNPs reported here were in HWE. We did initially find a single SNP that was out of HWE at 16q22.1 that had a highly significant for two-point logarithm of the odds (LOD) score of 7.76. This SNP had an excess of heterozygotes (approximately 70%) and a decrease in both homozygotes. We later found that this SNP was within a known copy number variant (CNV), which is responsible for the heterozygote inflation. This SNP was removed from all analyses and is not reported as significant.

### Statistical analysis

#### Remapping and merger of the SNP and STS data sets

After cleaning, we merged the exome variant data with older microsatellite (sequence tagged site (STS)) data from previous linkage studies in the same population [13, 14, 28]. All genetic markers (SNPs and STSs) were mapped onto a common genetic map, the Rutgers Map version 3 for GRCh37 [29]. After merging, the entire data set consisted of 665 individuals from 64 extended families with 67,196 markers (399 STS) for analysis. Family-specific marker allele frequencies were estimated using a Monte-Carlo expectation maximization algorithm in sib-pair [23] and used in all linkage analyses.

#### Phenotype classification

A full description of the phenotyping has been previously described [14] but briefly families were eligible to be included in the study if there was an index case with a spherical equivalent (SpEq) of -1D or lower and no systemic or ocular disease. All adults in the family were classified as affected or unaffected based on these same criteria. In children, a more stringent approach to classification was used in order to account for normal refractive development. Individuals between 6 and 10 years of age were classified as unaffected if their MSE in both eyes was +2D or higher, and individuals whose MSE was between +2D and -1D were designated as unknown. Individuals aged 11–20 years with a minimum MSE of + 1.5D in both eyes were classified as unaffected. In this age group, individuals with a MSE between + 1.5D and -1D were placed in the unknown class.

### Two-point linkage analyses

Two-point linkage analyses were performed using the program TwoPointLods [30]. This is a parametric linkage analysis program, and we assumed an autosomal dominant model with a disease allele (D) frequency of 0.0133 and a 90% penetrance and 10% phenocopy rate (dd/Dd/DD = 0.1/0.9/0.9). Analysis was performed individually on each family. Cumulative LOD scores and heterogeneity (HLOD) scores were then calculated across all families.

### SNP pruning for linkage disequilibrium

It is well-known that including markers that are in strong linkage disequilibrium (LD) in multi-point linkage analyses that assume linkage equilibrium can cause inflation of false positive rates. Previous analyses have allowed us to determine that even multi-point linkage analyses that attempt to adjust for intermarker LD are often inaccurate for very dense marker maps, so the data were pruned. All SNPs were condensed into 1 cM bins. The SNP with the highest minor allele frequency (MAF) in the bin was chosen to then represent the bin in the multi-point analyses. We performed further LD analysis on the binned SNPs in Haploview [31]. For any SNP-pairs with an r^2^ value greater than 0.2, one of the SNPs in the pair was removed. Because of their high information level, no STS markers were removed in pruning analyses. Thus, after cleaning we were left with 3764 markers.

### Multi-point linkage analyses

Multi-point linkage analyses were performed using SimWalk2 [32–34], with the identical models used in the two-point analyses.

### Collapsed haplotype pattern linkage analyses

A new approach to deal with intermarker LD without pruning is the collapsed haplotype pattern (CHP) method by Wang et al. [35] and implemented in the program SEQLinkage. This approach generates multiallelic pseudo-markers based on short haplotypes within specified genetic regions such as genes as determined using physical positions from RefSeq for GRCh37. The advantage of this approach it is does not require pruning as the multipoint analysis does. We then performed two-point linkage analysis of myopia with these pseudo-markers using Merlin [36].

### Association analyses

We also performed two types of association analyses. The family-based association test FBAT [37, 38] was used to examine all variants across all families. We also used rv-TDT [39] which examines rare variants (MAF < 0.05). We chose a single trio of genotyped individuals from each extended pedigree for this analysis.

### Functional annotation and microRNA target prediction

Variants were annotated using Annovar to get the most up to date predictions of function. Predicted microRNA targets were identified using miRanda [40] and scored using mirSVR [41].

## Results

Four samples were not released due to poor performance on the array. After quality control, there were 67,451 polymorphic variants and the mean call rate was 99%. Additional family members without DNA for genotyping were included to define family relationships. Demographic and clinical characteristics can be found in Table 1.

**Table 1:** Sample Demographics

Characteristics	Participants
*N*	665
Genotyped	582
Affection Status	
Affected	441
Unaffected	138
Unknown	86
Spherical Equivalent	
Mean	−3.46
Standard Deviation	3.29
Sex	
Male	343
Female	322

A summary of the four genome-wide significant chromosomal regions identified by either the two-point or CHP linkage analyses can be found in Table 2. Suggestively linked regions can be found in Additional file 2: Table S2.

**Table 2:** HLOD Scores for Genome-wide Significant Chromosomal Regions

Chr	cM	Highest SNP (TP) or Gene (CHP)	Max LOD Score	Max HLOD Score	Alpha for HLOD	Max Multipoint HLOD near this location	Alpha for Multipoint HLOD	Max CHP HLOD at this location	Alpha for CHP HLOD
1p36.12^a^	47.64	rs12748456^b^	**4.47**	**4.47**	1	1.21	0.15	**3.49**	0.48
8q24.22	147.50	rs72731540^c^	**3.75**	**3.75**	1	1.07	0.15	0.43	0.12
11p15.1^d^	30.74	*NCR3LG1*	1.27	2.78	0.55	0.27	0.05	**3.66**	0.57
7q36.1	161.66	*SSPO*	0.40	0.40	1	0.90	0.15	**3.92**	1

^a^ Multiple genome-wide significant two-point scores around this location. Also contained a single significant CHP variant, the *LINC00339* gene

^b^ Intergenic variant located between *LINC00339* and *CDC42*

^c^ Coding variant in *WISP1*

^d^ Multiple suggestive CHP scores in addition to the significant CHP score at this location

This table describes the four chromosomal regions that contained at least one significant HLOD score in either the two-point or CHP linkage analyses. Column 1 shows the chromosomal region that was found to be significant and column 2 shows the position of the region in centimorgans. Column 3 reports the location of the highest HLOD score in the region. If the highest HLOD was in the two-point (TP) analysis, a SNP rsID is reported; if the highest HLOD occurred in the CHP analysis, a gene name is reported instead. Columns 4–6 report the maximum cumulative LOD score, HLOD score and associated alpha for the two-point analysis, columns 7–8 show the maximum multipoint HLOD closest to this location and its associated alpha, and columns 9–10 display the maximum CHP HLOD at this location and its associated alpha. The overall highest HLOD score for each region is shown in bold

**Fig. 1:**
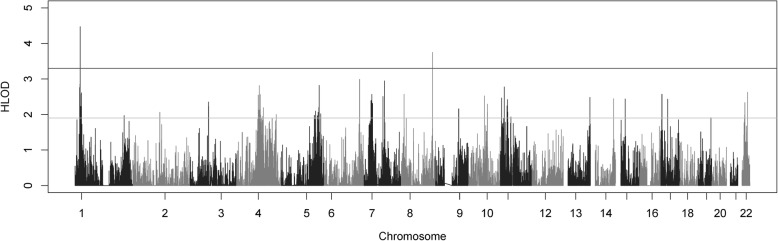
Genome-wide plot of two-point heterogeneity LOD (HLOD) scores across the 64 Ashkenazi families. The lines at 1.9 and 3.3 represent the respective suggestive and significant thresholds recommended by Lander and Kruglyak

**Fig. 2:**
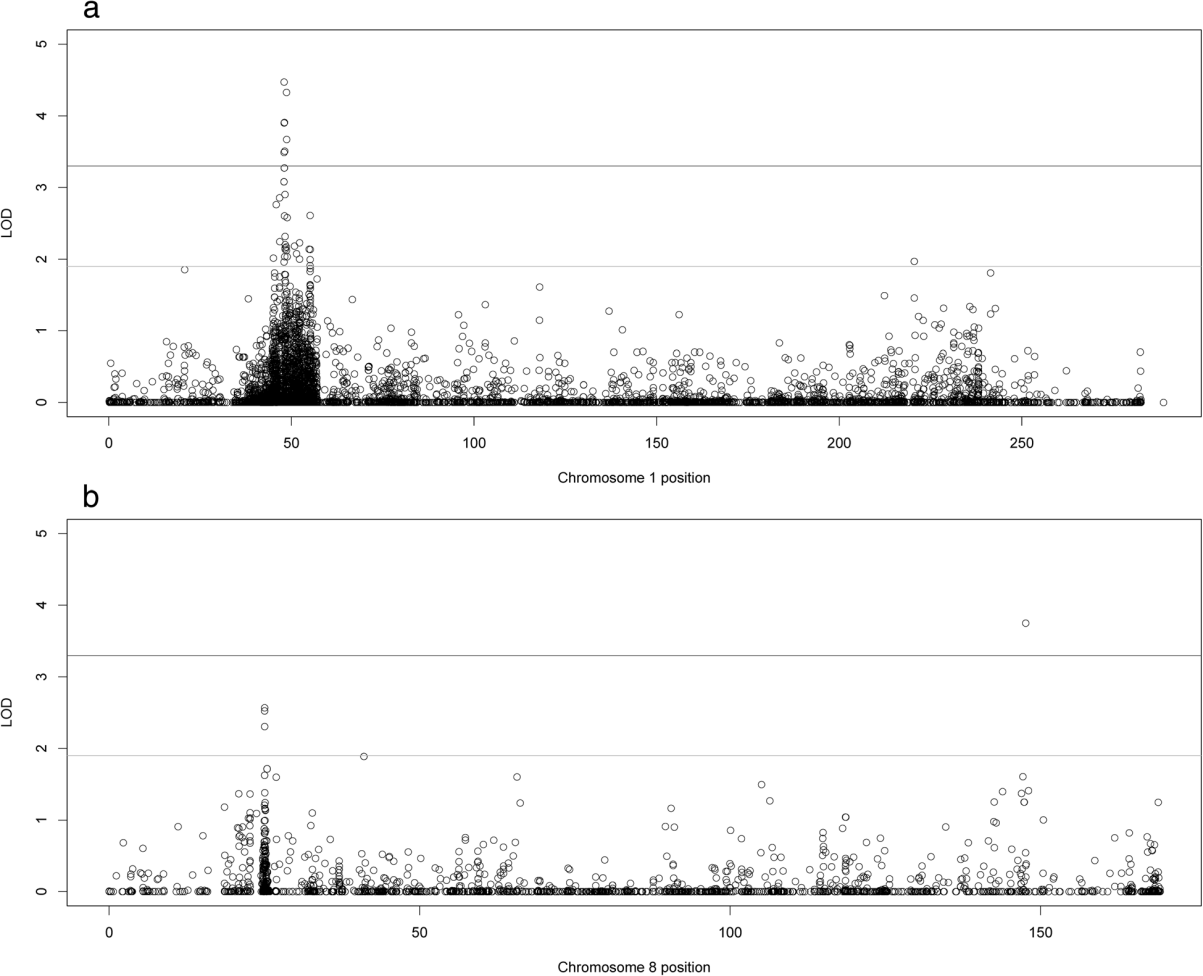
Chromosomal plots of two-point heterogeneity (HLOD) scores produced across the 64 Ashkenazi families. **a**. Plot of chromosome 1 in cM. **b**. Plot of chromosome 8 in cM. In both plots, the lines at 1.9 and 3.3 represent the respective suggestive and significant thresholds recommended by Lander and Kruglyak

**Fig. 3:**
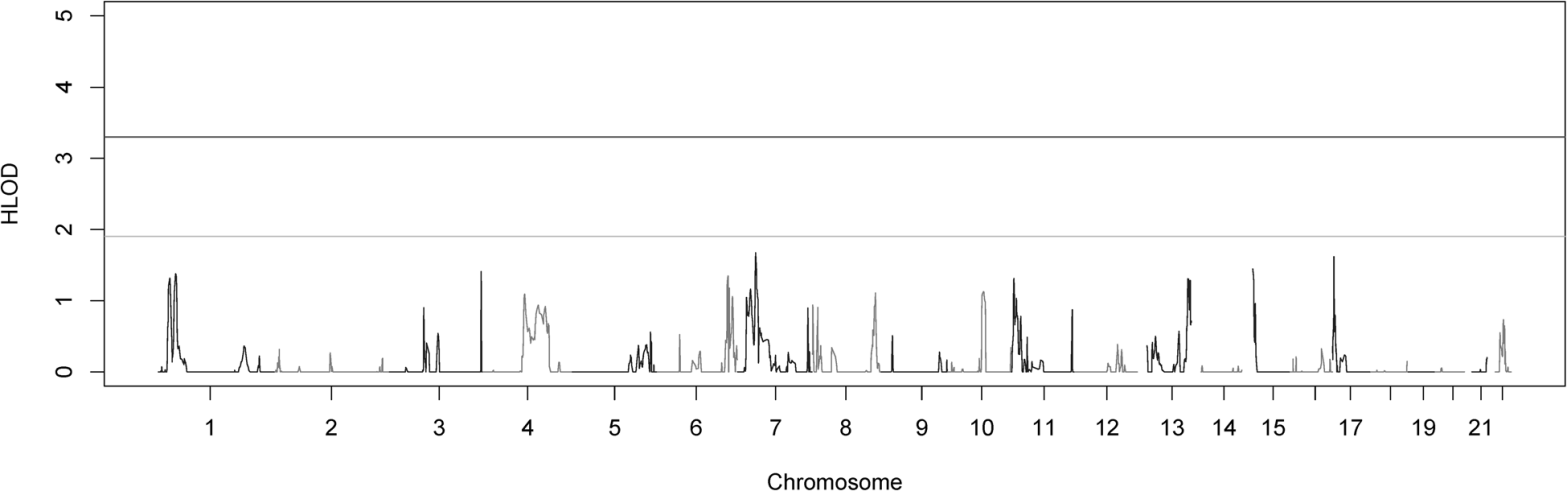
Genome-wide plot of multipoint heterogeneity LOD (HLOD) scores across the 64 Ashkenazi families. The lines at 1.9 and 3.3 represent the respective suggestive and significant thresholds recommended by Lander and Kruglyak

Two-point parametric linkage analysis was performed genome-wide (Fig. 1) and compared to previous multipoint linkage analysis both study-wide and on a family-by-family basis. Overall LOD scores and heterogeneity LOD (HLOD) scores were calculated. Seven genome-wide significant HLOD scores were observed for variants at chromosome 1p36.12 (max two-point LOD = 4.47). HLOD values > = 3.3 are considered significant and HLOD > = 1.9 are considered suggestive, as advised by Lander and Kruglyak [42] Multiple suggestive variants are also observed at 1p36 and a detailed plot of the two-point HLOD scores on chromosome 1 is provided in Fig. 2a. This region at 1p36.12 has previously been linked to ocular refraction but not myopia in these families [28] using multipoint analyses and a sparser set of markers. There was a single significant variant at chromosome 8q24.22 (max two-point LOD = 3.75) (Fig. 2b). Suggestive evidence of linkage was seen on multiple chromosomes, including 6 suggestive variants in the 11p15–13 region (Additional file 3: Table S3). Functional annotations of all SNPs with HLODs over 1.9 from wANNOVAR [43–45], along with the corresponding LODs and HLODS are shown in Additional files 4, 5, 6 and 7: Tables S4–S7. Multipoint linkage analyses of a pruned subset of SNPs using Simwalk2 did not produce as strong HLOD scores at these locations, even when specifically selecting SNPs that were significantly linked in the two-point analysis (Fig. 3). How much of this loss of signal is due to low information for linkage due to the sparse map produced by LD pruning is not clear. The significant signals on chromosomes 1 and 8 were no longer even suggestive. The two-point linkage signals are however still considered to be significant evidence of replication for a locus, by classic thresholds [42], although this cannot be considered a true replication of the 1p36.12 region linkage since this locus has been seen before in this dataset, albeit in a different but related trait and with a different set of markers.

**Fig. 4:**
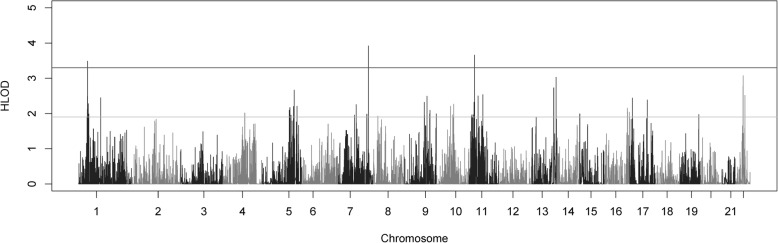
Genome-wide plot of genome-wide collapsed haplotype pattern heterogeneity LOD (HLOD) scores across the 64 Ashkenazi families. The lines at 1.9 and 3.3 represent the respective suggestive and significant thresholds recommended by Lander and Kruglyak

**Fig. 5:**
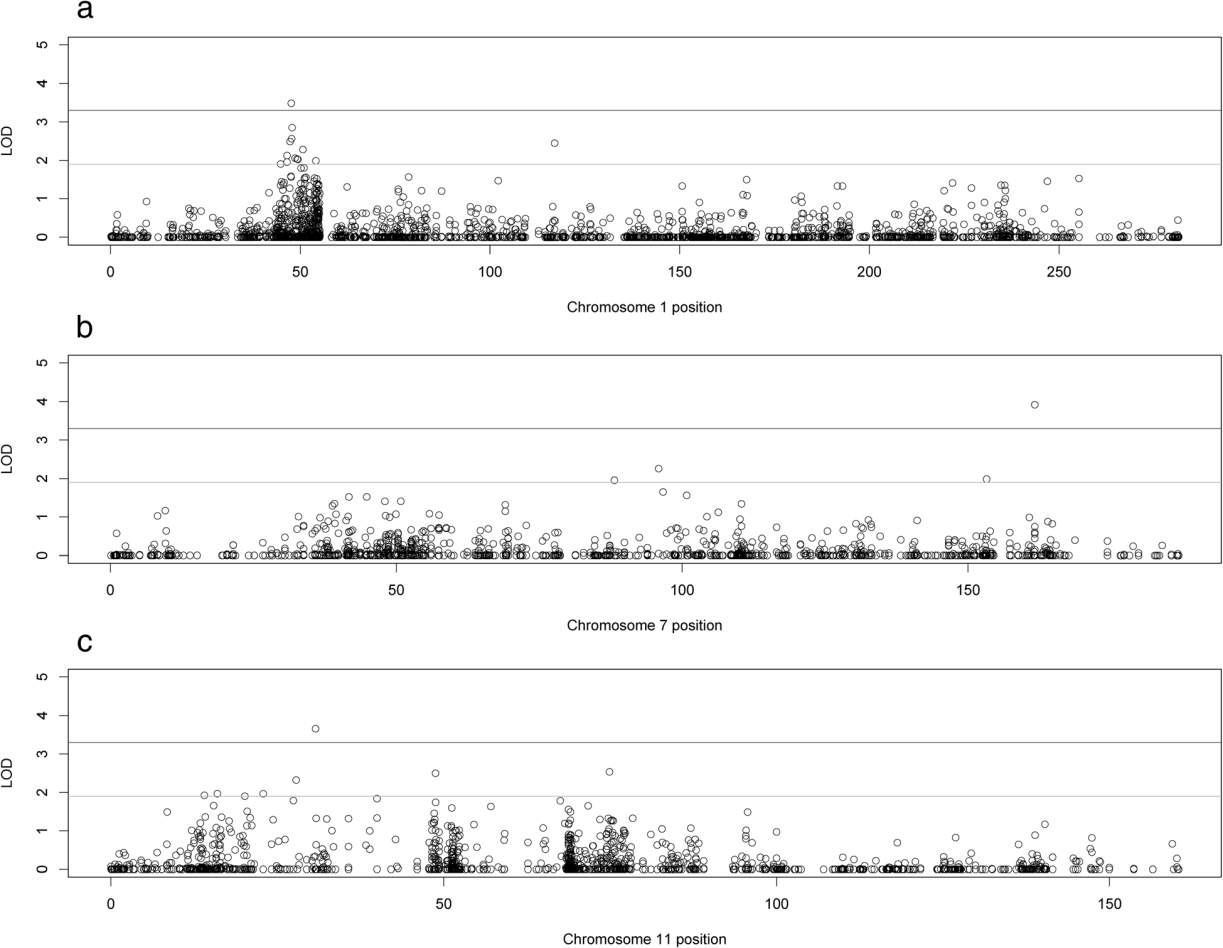
Chromosomal plots of CHP heterogeneity LOD (HLOD) scores produced by Merlin and SEQLinkage across the 64 Ashkenazi families. **a**. Plot of chromosome 1 in cM. **b**. Plot of chromosome 7 in cM. **c**. Plot of chromosome 8 in cM. In all plots, the lines at 1.9 and 3.3 represent the respective suggestive and significant thresholds recommended by Lander and Kruglyak

Collapsed haplotype pattern (CHP) linkage analysis of these data using SEQLinkage and Merlin identified three genome-wide significant genes. The first significantly linked gene was *LINC00339* (Fig. 4), in the 1p36.12 region (max HLOD 3.49, α = 0.48), which overlaps with the two-point linkage results seen above. This was the only significant gene found in the 1p region by the CHP analysis. The other two significant linkage signals were unique to this analysis. One was from the *SSPO* gene on chromosome 7q36.1 (max HLOD 3.92, α = 1), which does not overlap with the published chromosome 7p15 myopia locus *MYP17*, which is on the opposite chromosomal arm. The third significant linkage was to the *NCR3LG1* gene on chromosome 11p15.1 (max HLOD 3.66, α = 0.57). This latter linkage is within the same region as the suggestive 11p linkage observed in the two-point linkage analysis above. *NCR3LG1* is about 14 kb from *PAX6*. *PAX6* itself did not have any HLOD scores above 0.6 in the CHP analysis (although there was one suggestive two-point linkage to one *PAX6* variant with a maximum HLOD = 2.01). Suggestive CHP linkage was found at 11p14.1 (*BDNF*, maximum HLOD 2.50) and 11p15.2 (PDE3B, maximum HLOD = 2.32). (Additional file 3: Table S3). Individual plots of the CHP HLODs scores along chromosomes 1, 7, and 11 can be found in Fig. 5.

The association analyses using FBAT and rv-TDT found no genome-wide significant signals.

## Discussion

Here we report significant linkage with myopia at 1p36.12, 8q24, 7q36.1, and 11p15.1. The loci on chromosomes 1 and 11 are replications, while the loci on chromosomes 8 and 7 are novel. All of these linkage signals are cumulative effects across families. However, the families do not share identical linked haplotypes; if they did we should have seen significant association within these regions as well. This suggests that several different causal variants may exist across the linked families, with these causal variants possibly all being in the same gene (allelic heterogeneity).

Our strongest signals occurred in the 1p36.12 region, identified as significant in both the two-point and CHP analyses. Linkage of refractive error (but not myopia) to markers on 1q36 [28] has been reported before in this population, and is therefore not a true replication. It now appears that this region did not exhibit significant linkage with myopia in previous analyses due to prior insufficient marker information at this location to detect linkage to the binary trait of myopia affection. The 1p36.12 region contained the highest overall two-point and CHP HLOD scores, both located either in or near *LINC00339*, a long non-coding RNA gene known to be associated with endometriosis [46–48] but with no published role in ocular disease. *CDC42*, a GTPase directly downstream of *LINC00339*, contained three significant variants in the two-point analysis, but has not previously been implicated in myopia either. However, one of its activation targets, *LAMA1*, has been found to cause myopia in the presence of other phenotypes [49]. Slightly further upstream at 1p36.2, the genes *FRAP1* and *PDGFRA* (both located on 1p36.2) have both been found to be associated with corneal curvature and eye size in Asian and European populations [50, 51]. Neither gene was found to be even suggestively linked to myopia in this study.

We report the discovery of a novel locus linked to myopia on 7q36.1, distinct from another known chromosome 7 locus, *MYP17* [52–54], located on the opposite arm at 7p15. This locus was only detected by the CHP analyses and localized to the SCO-spondin gene (*SSPO*). The subcommissural organ (SCO) is one of the circumventricular organs, a set of brain structures that form the linkage between the central nervous system and the peripheral blood stream. It is one of the first differentiated brain structures to form and its function is largely unknown. SCO-spondin is a large glycoprotein from the thrombospondin. This protein is highly expressed during CNS development and is believed to be important in cellular adhesion, axonal pathfinding and homeostasis. The *Pax6* mutation which causes a small eye and is known as *Sey* also causes abnormalities in the SCO [55]. Homozygous *Sey/Sey* mice die at birth with numerous defects including an inability to properly form the SCO and *Sey/+* mice demonstrate a mosaic of SCO cells, some of which are not expressing the Reissner’s fiber, a fibrous aggregation of the secreted molecules of the SCO and is formed by secretion of SCO-spondin, and other abnormalities related to normal development of this important brain region. This admittedly tenuous link to *PAX6* is an intriguing addition to the complex story of myopia and its relationship to early brain development.

**Fig. 6:**
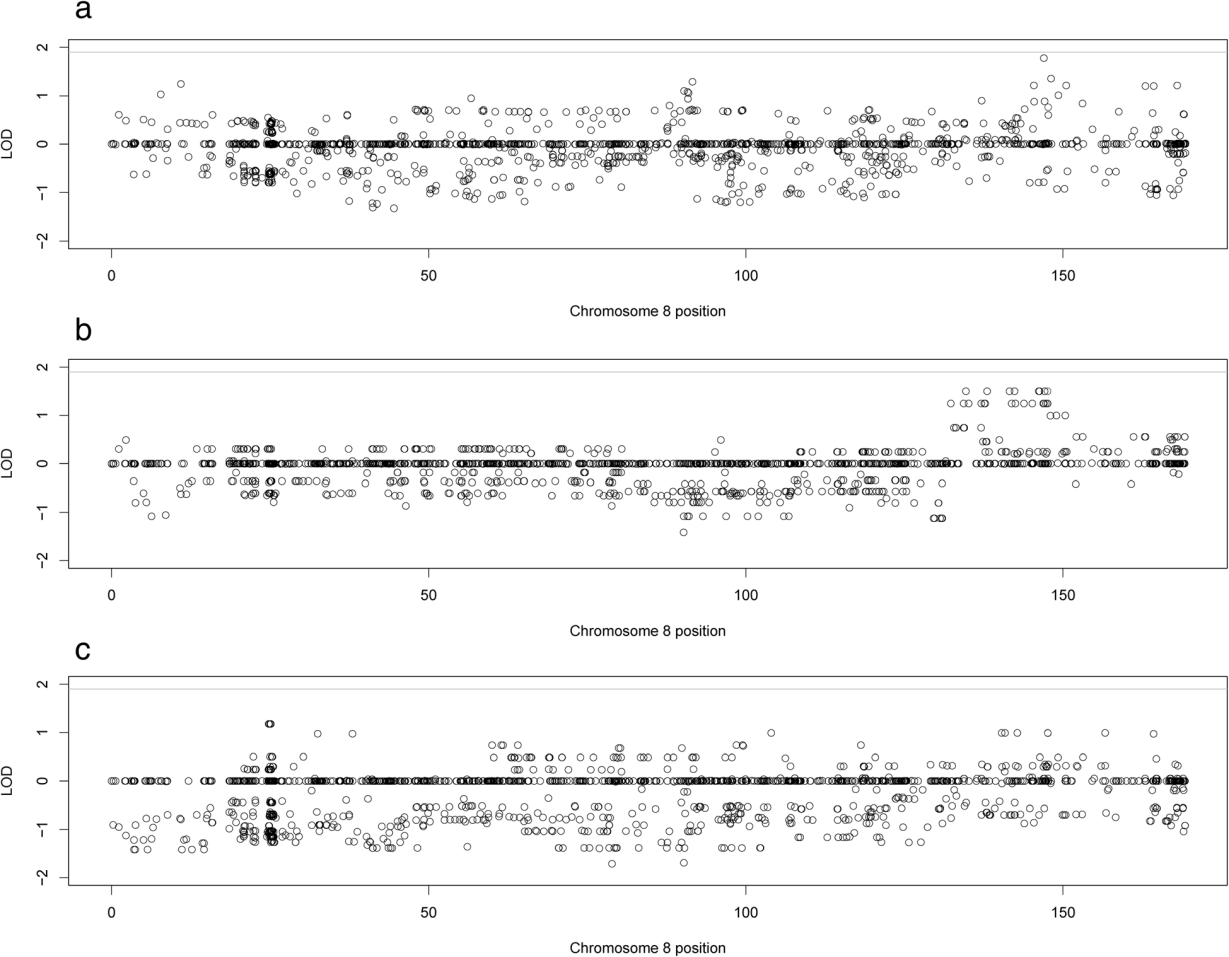
Plots of two-point LOD scores for across chromosome 8 (in cM) produced by TwoPointLods for the three strongest linked families. **a**. Plot of family 1019. **b**. Plot of family 1057. **c**. Plot of family 1068. In all three plots, the genome-wide significant nonsynonymous exonic variant located in *WISP1* is shown in blue and the line at 1.9 represents the suggestive threshold recommended by Lander and Kruglyak

A second novel locus discovered by two-point analysis on chromosome 8q24 has not been previously reported and the variant with the strongest two-point LOD score is located in the *WNT1*-inducible pathway protein 1 (*WISP1*). This gene is a member of the *WNT1*-inducible signaling pathway family of genes, all of which belong to the connective tissue growth factor family. It is a downstream regulator in the Wnt/Frizzled signaling pathway, is associated with cell survival by attenuating p53-mediated apoptosis in response to DNA damage through activation of AKT kinase and is widely expressed in many tissues. No prior eye disease associations currently exist for this gene, but the Wnt pathway is important in development of the eye. Significant linkage was only reported on a single variant in the two-point analysis, and this region was not significantly linked to myopia in either the multipoint or the CHP analyses. Thus it is possible that this is a false-positive two-point signal. However, linkage for this variant is driven by three families with LOD scores of 1.5, 1.0, and 0.76 and in each of those families the variant is part of a small linked haplotype across the 8q24 region, making it less likely that the signal is a false positive (Fig. 6). The variant itself has not been well studied; it does not have an rs ID and does not have any frequency information in 1000Genomes or ExAC for any population. In our dataset, the variant has a frequency of 0.047 and is nonsynonymous exonic.

Another replicated locus on 11p15 was present at a suggestive level in the two-point analysis but at the genome-wide significant level in the CHP analysis. This is a true replication as this signal has not been previously seen in this population and adds to the body of evidence that some genomic feature in this location appears to be actively modulating the risk of developing myopia and refractive errors. This signal overlaps with the *MYP7* locus [56] which spans 11p13-p15.4 and there is suggestive evidence of linkage in the two-point analyses of a 3’ UTR variant in *PAX6*. The role of *PAX6*, long postulated as a potential modifier of myopia risk, remains murky, with evidence both supporting and rejecting its involvement [17, 22, 57–66]. It remains to be seen whether repeated detection of signals in this location by multiple studies will turn out to be from *PAX6* or another nearby gene. The CHP analyses of these data by contrast localized the signal to another gene, *NCR3LG1* which is considerably upstream of *PAX6* but still within the linkage region originally identified by Hammond [17]. *NCR3LG1* is a natural killer (NK) cell cytotoxicity receptor ligand and when it interacts with NKp30 results in NK activation and cell death. It interacts exclusively with *NCR3* but not with other NK cell activating receptors. It has only been reported as expressed in tumor tissues. None of these facts make *NCR3LG1* a particularly attractive candidate for myopia development however, and there are many other candidate genes in the region that, based on biological function, may be more likely to be causal genes (Additional file 3: Table S3).

Although they did not reach genome-wide significance, it is interesting to note that several loci did meet the criteria for suggestive evidence of linkage, including 7p14 close to the *MYP17* locus at 7p15 [52–54].

It is unfortunate but not surprising that none of the association analyses were able to detect associations in the regions found in the linkage analyses. Family-based association analysis relies on risk alleles being shared across families either identical by state (IBS) or identical by descent (IBD). Linkage by contrast tracks the co-segregation of haplotypes and the trait within a pedigree, but is not concerned with whether those segregating haplotypes contain alleles IBS across different families. Using a founder population such as the Orthodox Ashkenazi Jewish families in this analysis increases the likelihood that there may be shared risk alleles across linked families, but this is not guaranteed. Therefore, this result, combined with the annotation of the significantly linked variants/genes discussed above, suggests that even using the exome-targeted array, we have likely not genotyped the actual causal allele(s) and instead are only able to detect its presence via linkage to specific haplotypes in each linked family.

## Conclusions

This study found significant linkage to myopia in Ashkenazi Jewish families at four chromosomal loci - 1p36.12, 8q24, 7q36.1, and 11p15.1. The signals at 7q and 8q were novel, while the signals at 1p and 11p are replications of previously identified signals, albeit ones where the causal genes have yet to be identified. We were able to identify several potential causal genes, including *WISP1* on 8q and *SSPO* on 7q, though with our limited exome-based array we were unable to resolve the signal further than the chromosomal regions. We plan to perform either targeted sequencing on the regions of interest or whole genome sequencing (WGS) on the most highly linked families to unequivocally identify the causal variants that account for the linkages to myopia detected here.

## Additional files

Additional file 1: **Table S1.** SNPs not released due to technical failure. A detailed list of the number of SNPs that failed at different points of the technical quality control. (XLSX 10 kb)

Additional file 2: **Table S2.** Two-point, Multipoint and CHP LODs and HLODs for Genome-Wide Suggestively Linked Regions (See Table 2 for Genome-wide Significantly Linked Regions). A list of the suggestively linked regions and their HLOD scores and alpha values for the three sets of parametric linkage analyses. (XLSX 11 kb)

Additional file 3: **TableS3.** List of known genes in genome-wide significant region on chromosome 11. A complete list of genes in the 11p region that was identified as significantly linked in the linkage analysis. (XLSX 44 kb)

Additional file 4: **Table S4.** Annotation of Suggestive and Significantly Linked Variants Sorted by Chromosome and then by HLOD - Two Point Linkage Results. A list of all variants that were either significantly or suggestively linked, along with annotations provided by annovar. (XLSX 43 kb)

Additional file 5: **Table S5.** Annotation of Suggestive and Significantly Linked Variants in the 1p36 region sorted by base pair start location - Two Point Linkage Results. A list of all variants on 1p36 that were either significantly or suggestively linked, along with annotations provided by annovar. (XLSX 15 kb)

Additional file 6: **Table S6.** Annotation of Suggestive and Significantly Linked Variants on chromosome 8 sorted by base pair start location - Two Point Linkage Results. A list of all variants on chromosome 8 that were either significantly or suggestively linked, along with annotations provided by annovar. (XLSX 11 kb)

Additional file 7: **Table S7.** Annotation of Suggestively Linked Variants on chromosome 11 sorted by base pair start location - Two Point Linkage Results. A list of all variants on chromosome 11 that were either significantly or suggestively linked, along with annotations provided by annovar. (XLSX 11 kb)

## Abbreviations

CHP: collapsed haplotype pattern
CIDR: Center for Inherited Disease Research
CNV: copy number variation
D: diopters
GC: genotype quality
GWAS: genome-wide association study
HLOD: heterogeneity logarithm of the odds
HWE: Hardy-Weinburg equilibrium
IBD: identical by descent
IBS: identical by state
LD: linkage disequilibrium
LOD: logarithm of the odds
MAF: minor allele frequency
MSE: mean spherical equivalent
NK: natural killer
SNP: single nucleotide polymorphism
SpEq: spherical equivalent
STS: sequence tagged site (microsatellite)
WES: whole exome sequence
WGS: whole genome sequence
